# La-Doped ZnTiO_3_/TiO_2_ Nanocomposite Supported on Ecuadorian Diatomaceous Earth as a Highly Efficient Photocatalyst Driven by Solar Light

**DOI:** 10.3390/molecules26206232

**Published:** 2021-10-15

**Authors:** Ximena Jaramillo-Fierro, Silvia González, Francesc Medina

**Affiliations:** 1 Departament d’Enginyería Química, Universitat Rovira i Virgili, Av. Països Catalans 26, 43007 Tarragona, Spain; francesc.medina@urv.cat; 2 Departamento de Química y Ciencias Exactas, Universidad Técnica Particular de Loja, San Cayetano Alto, Loja 1101608, Ecuador; sgonzalez@utpl.edu.ec

**Keywords:** TiO_2_, ZnTiO_3_, La-doping, heterojunction, absorption, photocatalysis

## Abstract

Currently, there is great interest in the use of TiO_2_ for photocatalytic remediation of wastewater. Doping, heterojunction, and immobilization on porous materials are effective methods to improve the photocatalytic efficiency of this semiconductor oxide. In this study, ZnTiO_3_/TiO_2_ (ZTO) and ZnTiO_3_/TiO_2_/La (ZTO/La) nanocomposites were successfully prepared and immobilized on diatomaceous earth (DE). The composition and texture of the composites prepared were characterized by X-ray diffraction (XRD), X-ray fluorescence (XRF), diffuse reflectance spectroscopy (DRS), scanning electron microscopy (SEM-EDX), and specific surface area (SSA). The adsorption capacity and photocatalytic activity of the composites were determined via degradation of methylene blue (MB) in batch reactors. The materials evaluated were prepared in the shape of 0.2 cm (diameter) and 1.0 cm (length) cylindrical extrudates. The results indicate that the ZTO/La-DE composite exhibited higher efficiency for the removal of MB under solar irradiation than both ZTO-DE and DE. The pseudo-second-order model and the Langmuir isotherm model were better suited to explain the adsorption process. The highest degradation percentage of MB obtained was 96% after 150 min of irradiation. The results indicate that synthesized composite could be used for the removal of cationic dyes in wastewater.

## 1. Introduction

In recent years, water contamination by dyes has become one of the most important global concerns [1,2]. Currently, there are more than 10,000 types of commercial dyes available with an annual production of over 7 × 10^5^ tons [3], and with a considerable fraction discharged from industries such as textile, paper, plastic, leather, ceramics, cosmetics, pharmaceuticals, and food processing [4,5,6]. In particular, effluents from these industries are considered an important source of pollution that generates by-products that are dangerous to health, in addition to preventing the penetration of sunlight and delaying photosynthesis in aquatic systems [7].

Recently, various chemical, physical, and biological treatment methods have been developed for the removal of dyes from aqueous solutions. These treatments include adsorption; precipitation; coagulation–flocculation; reverse osmosis; photocatalysis; oxidation with ozone, chlorine, or hydrogen peroxide; electrolysis, the use of anion exchange membranes; biological treatment; and other processes [8,9,10]. Among these, adsorption of the dye using porous materials and photocatalytic degradation of the dye through the use of semiconductors have attracted extraordinary attention in the last two decades [11,12,13,14,15]. Compared to other processes, adsorption and photocatalysis allow some flexibility in terms of simplicity of design, ease of operation, and low cost, as well as producing contaminant-free effluents that can be suitable for reuse [16]. 

Adsorption is a process that can be performed by different mechanisms, including electrostatic interaction, a chemical reaction such as complexation, or an ion exchange between the adsorbate and the adsorbent. Furthermore, pore structure and adsorbent surface chemistry exert the greatest influence on the adsorption process, whereas pore size distribution affects the efficiency and selectivity of the process [17]. For the adsorption process to be efficient, the adsorbent materials must have large specific surface area, high adsorption capacity, and active sites on their surfaces. In addition, they must be environmentally friendly, highly efficient, inexpensive, regenerable, and available in large quantities [18]. Therefore, mineral species such as clays, zeolites, and diatomaceous earth, among others [19,20,21], are the most promising materials for this purpose.

Diatomaceous earth (DE) or diatomite are natural fossilized remains of unicellular aquatic algae called diatoms. DE belongs to the group of almost pure sedimentary silica rocks that is typically composed by 87–91% silicon dioxide (SiO_2_), with significant amounts of alumina (Al_2_O_3_) and ferric oxide (Fe_2_O_3_) [22]. DE can also present variable amounts of impurities such as mineral clays, salts (mainly carbonates), and organic matter [23]. Other properties that allow DE to be industrially valuable include a low conductivity coefficient, low density, high porosity, large surface area, high adsorption capacity, and excellent thermal resistance [24]. Diatomite has been widely used in acoustic and thermal insulation, and as a filter aid, pharmaceutical carrier, and adsorbent. Recently, the use of diatomite has also been reported to be an excellent support material in the preparation of solid catalysts [25], because it presents siloxane bridges and silanol groups, which are key reactive sites for various surface reactions [26]. Thus, diatomite is a promising candidate for industrial production due to its great versatility, easy physical separation, and low cost [24].

On the other hand, heterogeneous photocatalysis is a promising advanced oxidation process (AOP) for the treatment of contaminated wastewater because it allows for complete mineralization of different organic and inorganic compounds using semiconductor catalysts that are activated by natural or artificial light [27,28,29,30,31,32,33,34]. The photocatalytic activity of semiconductors is influenced by a wide variety of factors such as morphology, specific surface area, affinity and adsorption capacity of organic pollutants, intensity and spectral distribution of the illuminating light, and pH of the solutions, among others [35]. Among the many semiconductors, TiO_2_ nanostructures have drawn increasing interest in wastewater treatment [36,37] due to several attractive properties including high reactivity, chemical stability, high oxidative capacity, non-toxicity, and low cost [38,39,40,41]. However, TiO_2_ has some disadvantages that hinder its cost-effectiveness and applicability, such as a wide bandgap (~3.2 eV), which limits its activity to a small proportion of the solar spectrum in the ultraviolet region. Furthermore, TiO_2_ has low quantum efficiency due to the rapid recombination rate of photogenerated electron-hole pairs, and shows a high particle agglomeration effect that reduces the number of active sites [42,43]. Many promising methods have been applied to address these characteristic disadvantages. For example, immobilization of the semiconductor in porous supports (e.g., diatomite) has proved to be an effective method to prevent agglomeration. At the same time, it increases the surface area and facilitates recovery of the photocatalyst at the end of the process. Likewise, intensive research has been conducted to improve the photocatalytic efficiency of TiO_2_ through common pathways such as doping with metallic and non-metallic ions, noble metal deposition, sensitization by inorganic complexes or organic dyes, coupling of semiconductors, and doping with rare earth (RE) elements [44,45,46,47,48].

Doping has long been known as one of the most effective approaches for altering the intrinsic electron structure of TiO_2_, lowering its bandgap energy, and consequently enhancing its sensitivity to visible light by improving electron-hole separation or extending the optical absorption span [49,50]. Among various dopants, rare earth elements have received much attention for the preparation of versatile photocatalysts [51,52,53,54,55]. Lanthanum is a widely investigated rare earth metal element, and the efficacy of using La ≤ 1–2 wt.% to dope TiO_2_ enhances its photocatalytic activity in both the UV and visible-light region [56,57,58,59,60]. Since the ionic radius of the La^3+^ ion (1.03 Å) is much higher than that of Ti^4+^ (0.64 Å), La^3+^ would disperse on the surface of TiO_2_ particles, forming Ti-O-La bonds rather than replacing the lattice site of Ti^4+^, according to several studies [61,62,63,64]. Lanthanum-doped compounds are generally used as efficient catalysts and light-conversion devices due to their electronic, optical, and chemical characteristics arising from their 4f electrons transition [65,66]. In particular, doping TiO_2_ with La can inhibit phase transition from anatase to rutile and restrain crystal growth [67], increase the surface area and the concentration of surface hydroxyl (-OH) groups, improve the optical properties due to the increase in the concentration of oxygen vacancies, and promote chemical adsorption of the organic substrates on the semiconductor’s surface, which also benefits the improvement of its photocatalytic efficiency for diverse applications [68,69,70,71,72,73,74,75]. 

Like the doping process, the coupling or heterojunction of two semiconductors that possess different levels of redox energy for their corresponding conduction (CB) and valence (VB) band has been extensively studied. This coupling has proven to be an attractive approach to compensate for the disadvantages of individual components and lead to more efficient charge separation, longer life of charge carriers, and improved interfacial charge transfer to adsorbed substrates [76,77]. Several semiconductors have been reported for the potential coupling of TiO_2_, including SiO_2_, MoO_3_, CdS, MgO, WO_3_, SnO_2_, ZrO_2_, CuO, Fe_2_O_3_, and ZnO [78,79,80,81]. The characteristics and compatibility of the coupling semiconductor are important for the physicochemical properties and stability of the hybrid semiconductor. Each semiconductor substantially affects the surface charge of the material and therefore increases or weakens its photocatalytic capacity [30]. Among the numerous semiconductor combinations, the integration of ZnTiO_3_ with TiO_2_ has previously been reported as a promising alternative for the adsorption and photocatalytic degradation of MB in wastewater [82,83]. Although ZnTiO_3_ has proven to be the most versatile perovskite-type oxide for various applications [84], the physical and chemical properties of the ZnTiO_3_/TiO_2_ heterojunction have been shown to be greater than those of the individual components, evidently resulting from the modification of their electronic states [85].

This paper reports on the doping of the ZnTiO_3_/TiO_2_ nano-heterojunction with lanthanum that was synthesized using the sol-gel method. Nanocomposites of ZnTiO_3_/TiO_2_ (ZTO) and ZnTiO_3_/TiO_2_/La (ZTO/La) were immobilized on diatomaceous earth (DE) to achieve innovative and eco-friendly nanomaterials, with adsorbent and photocatalytic properties for the effective removal of methylene blue (MB) in wastewater. The adsorption and photocatalytic degradation of MB were determined in batch experiments. The dye amount was determined by UV-visible spectrophotometry. The adsorption capacity of the synthesized composites was measured by varying the pH of the solutions, the concentration of the adsorbent, and the contact time, whereas the photocatalytic activity was determined under solar irradiation. The synthesized composites were characterized using X-ray diffractometry (XRD), X-ray fluorescence (XRF), diffuse reflectance spectroscopy (DRS), scanning electron microscopy (SEM-EDX), and specific surface area (SSA).

## 2. Results

### 2.1. Characterization of the Samples

#### 2.1.1. XRD and XRF Analysis

**Figure 1:**
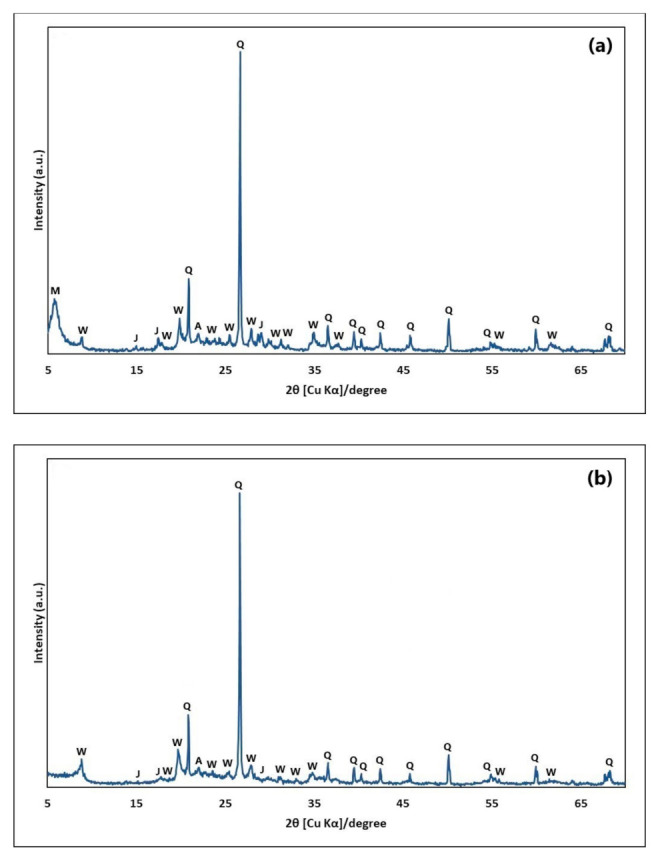
X-ray diffraction (XRD) pattern of diatomaceous earth (DE): (**a**) raw and (**b**) purified. Q: quartz, J: jarosite, A: albite, W: muscovite, M: montmorillonite.

Figure 1 displays the XRD pattern of diatomaceous earth (DE), (a) raw and (b) purified, which consists of quartz (Q), jarosite (J), albite (A), muscovite (W), and montmorillonite (M). By comparing Figure 1a,b, it can be noticed that the main peak related to montmorillonite at d-spacing = 15.0 (2θ = 6°) disappeared, probably due to the purification process.

In addition, the XRF analysis demonstrated that raw DE contains mainly SiO_2_ and Al_2_O_3_ as well as other oxides, which are shown in Table 1.

**Figure 2:**
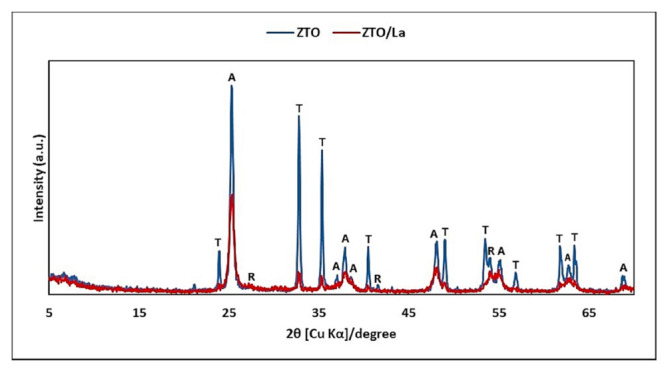
X-ray diffraction (XRD) pattern of ZnTiO_3_/TiO_2_ (ZTO) and ZnTiO_3_/TiO_2_/La (ZTO/La). T: titanate, A: anatase, R: rutile.

On the other hand, Figure 2 shows the XRD pattern of the ZnTiO_3_/TiO_2_ (ZTO) and ZnTiO_3_/TiO_2_/La (ZTO/La) nanocomposites. The characteristic peaks of ZnTiO_3_ were shown at 2θ~32.79° and 35.31°. The nanocomposite, in addition to the ZnTiO_3_ phase, consisted mainly of the anatase phase, whose characteristic peak appeared at 25.28° and, to a lesser extent, the rutile phase, whose characteristic peak appeared at 27.40° only for ZTO. This demonstrates that La-doping inhibited the transition of anatase to the rutile phase at the temperature–time conditions applied [2]. Furthermore, due to its low concentration, there were no diffraction peaks (50° and 60°) corresponding to the presence of La_2_O_3_ oxide [59].

The crystalline sizes of ZnTiO_3_/TiO_2_ doped with La (ZTO/La) were calculated based on the main peak using the well-known Scherrer equation (Equation (1)) [86,87].

(1) $$A=\frac{K\lambda }{\beta \;cos\theta }$$

where *K* is the shape factor (here, *K* = 0.89) and *λ* is the wavelength of the X-ray beam used (here, *λ* = 0.15406 nm, *θ* is the Bragg angle, and *β* is the full width at half maximum (FWHM) of the X-ray diffraction peak, which was calculated using the MDI JADE computer software, version 6 (Materials Data Inc., Livermore, CA, USA, 014)). The average crystalline sizes of the main phases present in the ZTO/La nanocomposite were 29.09 (±0.92) and 16.33 (±1.03) nm for ZnTiO_3_ and TiO_2_ (anatase phase), respectively. These values were lower than those calculated for the ZnTiO_3_/TiO_2_ nanocomposite: 41.35 (±1.27) and 26.76 (±1.31) nm for ZnTiO_3_ and TiO_2_ (anatase phase), respectively. From these results, the specific effect of lanthanum on the inhibition of crystallite growth and the stabilization of the ZnTiO_3_ and TiO_2_ phases was observed. 

#### 2.1.2. Optical and Photoelectric Properties

**Figure 3:**
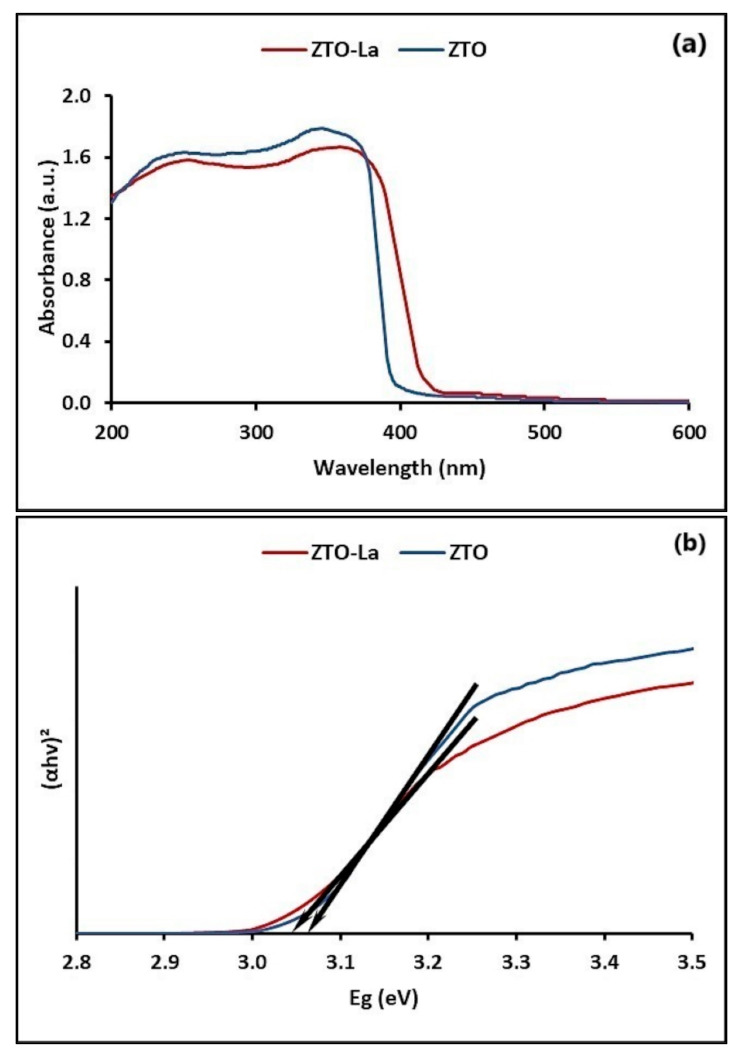
(**a**) UV-vis DRS and (**b**) plots of (αhv)^2^ vs. *E_g_* of ZnTiO_3_/TiO_2_/La and ZnTiO_2_/TiO_2_.

The optical absorption properties of photocatalysts can be characterized by the UV-visible (UV-vis) DRS in the range of 200–600 nm at room temperature. Figure 3a shows the UV-vis DRS of ZnTiO_3_/TiO_2_ (ZTO) and ZnTiO_3_/TiO_2_/La (ZTO/La). Comparatively, the visible light absorption intensity of the ZTO/La spectrum, at around 400 nm, was slightly improved, suggesting that the ZTO/La composite has better response to visible light. The graphs of (*αhv*)^2^ versus *hv* to calculate the direct band-gap energy (*E_g_*) are shown in Figure 3b. According to this figure, the direct *E_g_* values obtained from the intersections of the straight line with the energy axis [88] were 3.07 and 3.04 eV for ZTO and ZTO/La, respectively. The direct *E_g_* values, represented in Figure 3b, were calculated for ZTO and ZTO/La using the Equation (2) [89].

(2) $$E_{g}=\frac{1240}{\lambda }$$

where *E_g_* is the band-gap energy in electron volts (eV) and *λ* represents the lower cutoff wavelength in nanometers (nm).

#### 2.1.3. SEM and EDS Analysis

**Figure 4:**
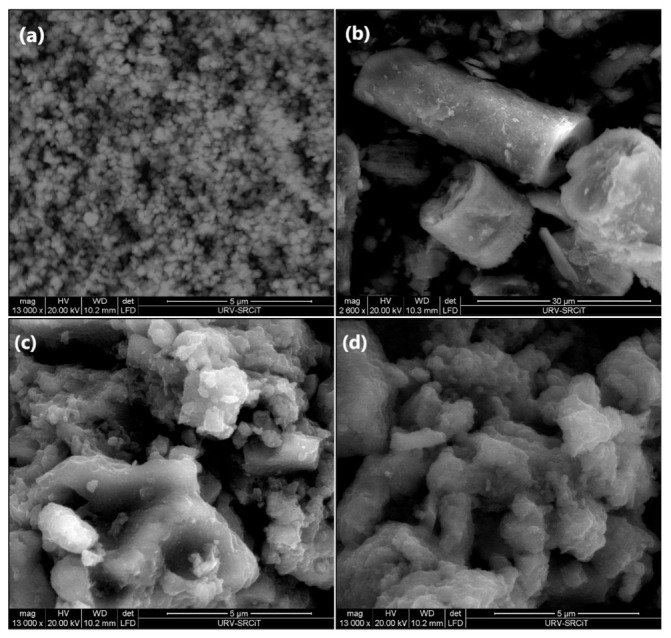
Scanning electron microscopy (SEM) images of (**a**) ZnTiO_3_/TiO_2_/La (ZTO/La), (**b**) diatomaceous earth (DE), (**c**) ZnTiO_3_/TiO_2_-DE (ZTO-DE), and (**d**) ZnTiO_3_/TiO_2_/La-DE (ZTO/La-DE).

Figure 4a shows the SEM micrographs of ZTO/La, which consisted of nearly spherical particles that had a strong tendency to form agglomerates. These particles were smaller in size than the non-doped nanocomposite that we reported in previous studies [90]. The mean particle size of the ZTO compound without the addition of La^3+^ was 98 nm, in contrast to the ZTO/La compound with La^3+^ ions, where the mean particle size was 78 nm. The results presented indicate that, as a dopant, La^3+^ is effective at hindering the growth of crystallites and stabilizing the ZTO compound. Additionally, the surface morphology of Ecuadorian diatomaceous earth (DE) was also investigated by SEM, and the results are shown in Figure 4b, from which it can be seen that the initial DE showed a cylindrical structure with a length of approximately 14–36 μm, an external pore diameter of around 16 μm, and an internal pore diameter in the higher cylinder of around 6 μm. There was a nearly regular array of submicron pores in an average diameter of 286 nm in the wall. Because of the macroporosity and the micron scales, reactants diffusion and physical separation are very facile [24]. The SEM micrographs of both ZnTiO_3_/TiO_2_-DE (ZTO-DE) and ZnTiO_3_/TiO_2_/La-DE (ZTO/La-DE) are presented in Figure 4c,d, respectively. In these figures, the supported composites appear with fewer cylindrical structures but with some catalyst particles incorporated (smaller ZTO and ZTO-La grains) on the outer face of DE.

**Figure 5:**
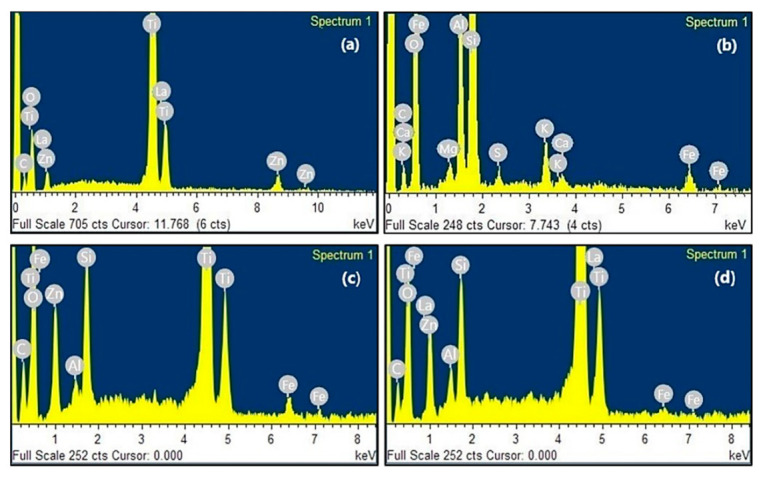
Energy dispersive X-ray (EDX) spectrum of (**a**) ZnTiO_3_/TiO_2_/La (ZTO/La), (**b**) diatomaceous earth (DE), (**c**) ZnTiO_3_/TiO_2_-DE (ZTO-DE), and (**d**) ZnTiO_3_/TiO_2_/La-DE (ZTO/La-DE).

The presence of La in the ZTO/La synthesized composite was confirmed by energy dispersive X-ray spectroscopy (EDS) (Figure 4a). According to the EDS analysis of the pure and La-doped composite, lanthanum was incorporated into ZTO nanoparticles. According to the EDS analysis, ZTO/La consisted of C (8.02%), O (59.29%), Ti (28.79%), Zn (2.47%), and La (1.43%). On the other hand, according to the EDS analysis in Figure 5b, DE consisted of C (11.62%), Ca (0.52%), K (2.19%), Fe (2.79%), O (50.41%), Mg (0.61%), Al (6.32%), Si (24.86%), and S (0.68%). The EDS analysis of Figure 5c,d confirmed that ZTO-DE- and ZTO/La-DE-supported composites contained an important amount of titanium and zinc, respectively, whereas lanthanum was present only in ZTO/La-DE. 

#### 2.1.4. Specific Surface Area (SSA) Analysis

The specific surface area of the adsorbents, both in their powder and extrudate forms, are summarized in Table 2. The extruded adsorbents prepared had a smaller surface area compared to that of adsorbents in powder form probably due to the heat treatment required for their preparation and to the lower surface area of DE. Despite the reduction in the specific surface area of the extrudates, the presence of exchange cations in their structure can contribute to elimination of the dye from the solution, since different mechanisms participate in the adsorption process.

### 2.2. MB Adsorption

#### 2.2.1. Effect of pH

**Figure 6:**
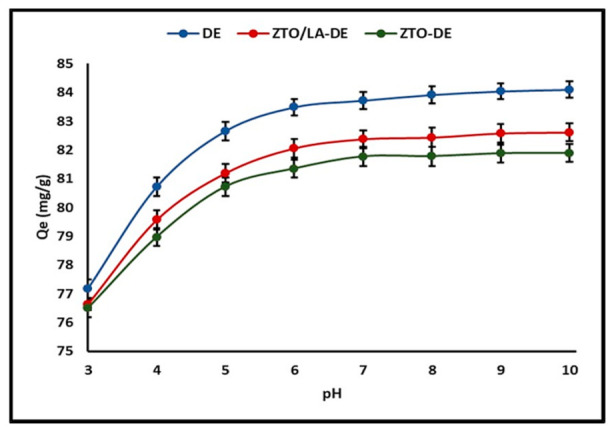
Effect of pH on MB adsorption onto composites.

DE showed a pH_PZC_ value of around 4.4, whereas the ZTO-DE and ZTO/La-DE extrudates showed pH_PZC_ values of around 6.2. At a pH higher than pH_PZC_, the surface had a net negative charge and adsorption of the cationic dye molecule was promoted. However, MB adsorption was reduced at a pH lower than pH_PZC_ due to the net positive charge on the surface, which caused electrostatic repulsion. Figure 6 shows this effect of pH on DE, ZTO-DE, and ZTO/La-DE extrudates.

From the minimal increment in MB adsorption in the solution at pH values above 8, it was decided that adsorption at pH = 7 was the optimum operating condition for adsorption experiments.

#### 2.2.2. Adsorption Isotherm

**Figure 7:**
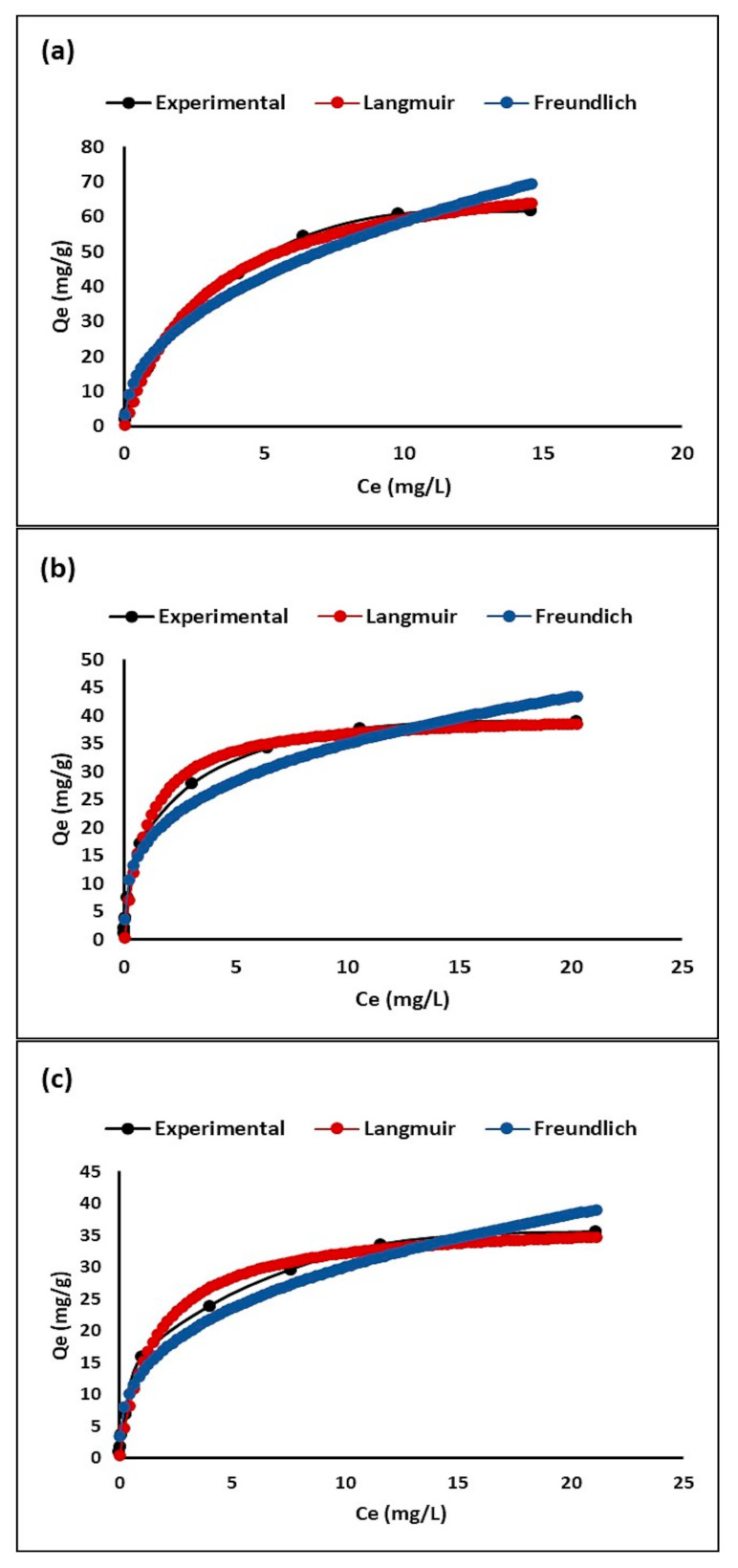
Adsorption isotherm of (**a**) DE, (**b**) ZTO/La-DE, and (**c**) ZTO-DE.

Figure 7 shows the adsorption isotherms of the extruded composites: DE, ZTO-DE, and ZTO/La-DE. This figure shows that the behavior of all composites fit the Langmuir model better than the Freundlich model.

Table 3 shows the equilibrium data of MB adsorption by extruded composites DE, ZTO-DE, and ZTO/La-DE. Furthermore, the R_L_ separation factor or equilibrium parameter was calculated using Equation (3), obtaining low *R_L_* values for all the adsorbents. When 0 < *R_L_* < 1, favorable adsorption was indicated, and *R_L_* > 1 meant unfavorable adsorption; *R_L_* = 0 indicated irreversible adsorption, and *R*_L_ = 1 meant energy dispersive X-ray linear adsorption [91]. 

#### 2.2.3. Adsorption Kinetics

**Figure 8:**
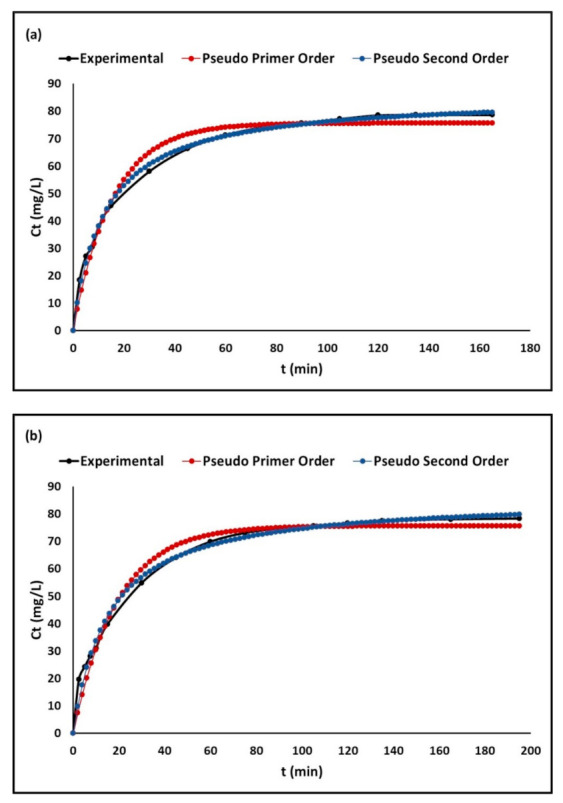

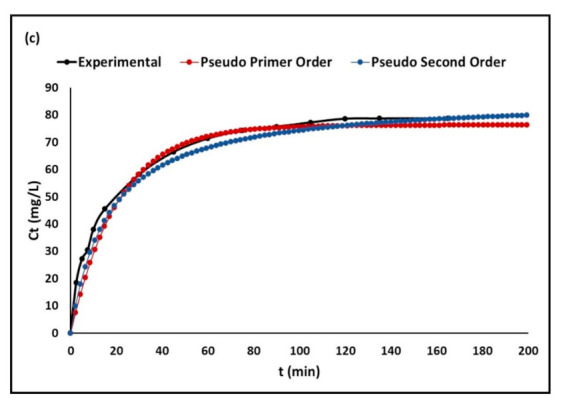
Adsorption kinetics of (**a**) DE, (**b**) ZTO/La-DE, and (**c**) ZTO-DE.

Figure 8 shows the time–course variation of the *C_t_* (mg/L) curves of the extruded composites: DE, ZTO-DE, and ZTO/La-DE. This figure indicates that the pseudo-second-order model was better than the pseudo-first-order model to describe the behavior of all composites. The figures show that the MB concentration in the solution decreased rapidly around the first 60 min, after which removal tended to become constant. 

**Figure 9:**
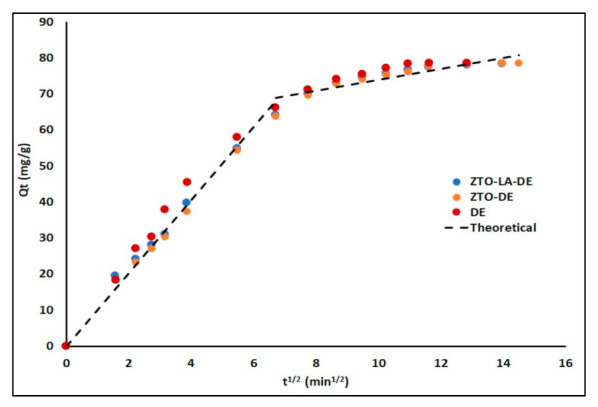
Intra-particle diffusion plots for MB removal by the extrudates.

The intra-particle diffusion in Figure 9 indicates that two steps occurred in the adsorption process. The initial and the second portions in each plot may have been products in the boundary layer effect and intra-particle diffusion, respectively. The initial steep-slope portion is attributed to external surface adsorption or instantaneous adsorption, whereas the relatively flat-slope portion followed by the initial portion can be attributed to the gradual adsorption stage where intra-particle diffusion was the rate-limiting step [59,92].

Table 4 shows the equilibrium data of MB adsorption by extruded composites DE, ZTO-DE, and ZTO/La-DE.

### 2.3. Photocatalytic Degradation of MB

**Figure 10:**
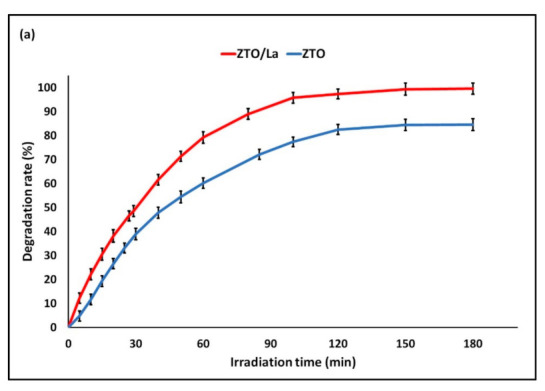

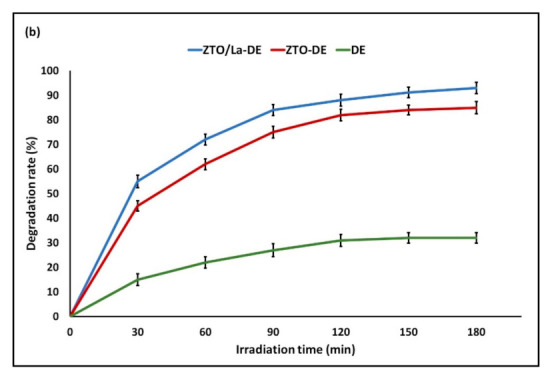
Photocatalytic degradation of MB by (**a**) a photocatalyst and (**b**) a supported photocatalyst.

Photocatalysts can efficiently decompose organic substances because of their strong oxidizing ability, which is generated when the photocatalysts are irradiated by light. In this paper, the photocatalytic activity of ZTO, ZTO/La, ZTO-DE, and ZTO/La-DE composites was tested by the decomposition of methylene blue (MB) in water using solar light. Figure 10 shows the results obtained in the test.

**Figure 11:**
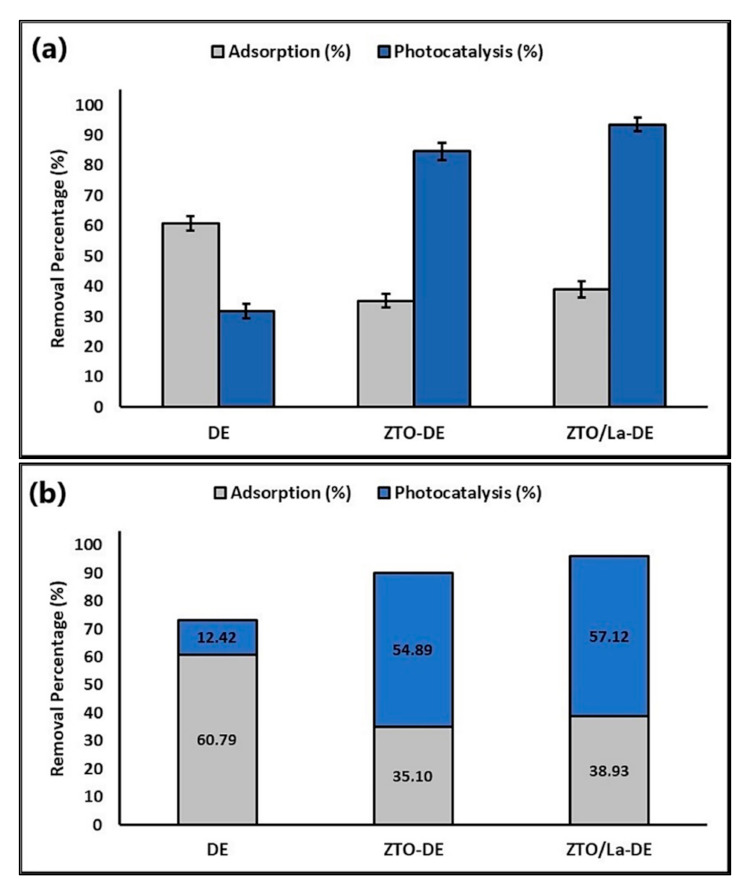
Percentage of (**a**) individual and (**b**) accumulated MB adsorbed and photodegraded by the composites.

Figure 11a shows that DE had a higher capacity for adsorption of the MB dye than supported semiconductors. Moreover, ZTO-DE and ZTO/La-DE had higher photocatalytic activity than DE. Figure 11b shows that DE-supported photocatalysts had higher efficiency for MB removal in aqueous systems.

### 2.4. Reuse of the Composites

**Figure 12:**
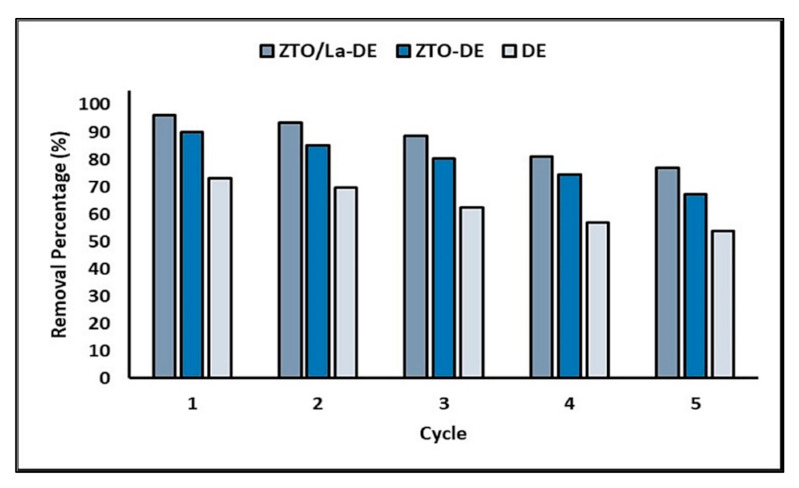
Percentage of MB removal during five successive adsorption–photocatalysis cycles.

As the stability and recyclability of the photocatalysts are considered important factors for their application on a large scale, five consecutive removal runs were carried out for the DE, ZTO-DE, and ZTO/La-DE extrudates. Figure 12 shows the efficiency (removal percentage) of these materials for five cycles.

Figure 12 clearly shows that the percentage of MB removal decreased slightly with increasing cycle times. However, after five cycles the synthesized materials still had high activity and could efficiently degrade MB in aqueous solution.

## 3. Discussion

### 3.1. Characterization of the Samples

#### 3.1.1. XRD and XRF Analysis

Figure 1 illustrates the XRD pattern of diatomaceous earth (DE), in which the high content of SiO_2_ in the form of quartz is clear, as well as other mineralogical phases consistent with the XRF results (Table 1). The chemical and mineralogical composition of DE is in accordance with that reported in the literature [26]. In Figure 2, it is observed that the La ion significantly reduced the intensity of the zinc titanate (ZnTiO_3_) and anatase peaks of the doped compound (ZTO/La). According to the literature, the La ion has an ionic radius of 1.03 Å and could therefore not replace Ti cations with an ionic radius of 0.64 Å, but could potentially be located on the surface of ZnTiO_3_ crystallites and anatase in small amounts [72]. The presence of Ti-O-La on the surface of the hybrid catalyst’s crystallites can contribute to the decrease in the intensities of the diffraction peaks [64]. This is because segregation of doping cations on the crystallites’ surface inhibits their growth by restricting direct contact with neighboring crystallites, which leads to the stabilization of small crystalline particles [60,67]. The rutile phase is not present in the doped compound, probably because the La ion would greatly delay transformation from the anatase phase to the rutile phase [93,94]. No diffraction peaks of lanthanide oxides in the ZTO/La patterns were observed. This is probably due to the low amount of La ions (~1%) and also to the fact that the lanthanide oxides would be well dispersed in the ZnTiO_3_ and anatase phases [55]. 

#### 3.1.2. Optical and Photoelectric Properties 

The UV-vis optical absorption spectra of ZTO and ZTO/La heterojunctions are shown in Figure 3a. With respect to the undoped ZTO, it is clear that the absorption threshold was slightly shifted to the visible light region. Various authors have also reported a red-shift in UV to visible light absorption caused by La^3+^ doping into TiO_2_ [47,95]. However, other authors have reported a blue-shift in the absorption profile of La-doped TiO_2_ [96] and even an unchanged absorption spectrum for La-doped TiO_2_ relative to pure TiO_2_ [97]. Furthermore, plots of (*αhv*)^2^ versus hv in Figure 3b reveal that the bandgap (*Eg*) values of ZTO and ZTO/La were estimated at 3.07 and 3.04 eV, respectively. The bandgap plays a critical role in the photocatalytic activity of photocatalysts due to the fact that it participates in determining the e^−^/h^+^ recombination rate [11]. From the result observed, the bandgap of ZTO decreased when it was doped with La^3+^. Therefore, it is shown that ZTO/La is more active than ZTO under solar irradiation, probably due to the lesser separation between occupied and unoccupied bands [34].

#### 3.1.3. SEM and EDS Analysis

The SEM photographs and EDX spectra in Figure 4 and Figure 5, respectively, confirm the immobilization of ZTO and ZTO/La in DE. Photocatalysts immobilized in DE were relatively uniform, with some agglomerations that could have been covering the characteristic DE skeletons. The use of DE to immobilize nanostructured semiconductors was an effective alternative to obtain porous photocatalysts with better active surface and adsorption capacity than isolated semiconductors, keeping their electronic and structural properties for their application in the MB degradation under solar irradiation.

#### 3.1.4. Specific Surface Area (SSA) Analysis

The specific surface area (SSA) was estimated by nitrogen adsorption at a low temperature (−196 °C). The result listed in Table 2 shows that ZTO/La had a higher specific surface area, around 126.45 m^2^/g, compared to the ZTO compound, whose specific surface area was 105.84 m^2^/g. The increase in the surface area of ZTO/La was probably due to the decrease in the size of the primary crystallites, as well as the different phase composition of these samples [98]. Table 2 also shows that the extrudates had a lower specific surface area than the powdered materials. The decrease in the surface area of the extruded adsorbents after heat treatment is essentially attributed to the elimination of the physically adsorbed water as well as to the surface hydroxyl groups loosely bound to the DE structure [99,100,101]. Dehydration creates additional spaces within the DE porous structure, which probably contracts, causing the internal surface area to decrease. The relatively high surface area of the DE-immobilized ZTO/La nanocomposite could be a promising material for adsorption and photocatalysis, as well as for other applications. In fact, preliminary studies on the adsorption capacity and photocatalytic activity of the ZTO compound showed promising results for methylene blue (MB) removal in aqueous systems under irradiation with ultraviolet light [83,90]. Although the powdered materials usually have a higher SSA, in this study, the extrudates were chosen to adsorb MB due to their appropriate mechanical and chemical stability, which facilitated their recovery at the end of the process and their reuse after several cycles.

### 3.2. MB Adsorption

Batch adsorption of MB was performed from an aqueous solution to investigate the adsorption properties of ZTO-DE, ZTO/La-DE, and DE. Although the extrudates showed a lower specific surface area, they were also effective in removing MB from the aqueous solution, probably through other mechanisms, including electrostatic interaction, chemical reactions such as complexation, or ion exchange between adsorbent and MB [82]. Consequently, despite the reduction in the specific surface area of the extrudates, the surface chemistry of these materials was also an important factor controlling MB adsorption. The extruded DE showed a pH_PZC_ value of around 4.4 and the adsorption tests were carried out at pH = 7.0; therefore, the surface of these materials was negatively charged, improving adsorption of the cationic dye. In addition, according to XRF, DE contained various cations, such as Mg, K, Ca, and Fe, that could promote the cation exchange capacity of the extruded composites prepared to improve their MB adsorption capacity [102]. Likewise, there are several parameters that determine the effectiveness of the adsorption process. In this study, experiments were developed by varying the following parameters: initial pH of the MB solution, initial MB concentration, and contact time.

#### 3.2.1. Effect of pH

During the adsorption process, pH can affect the surface charge of the adsorbent, the electrical charge of the dye, and the degree of ionization. Adsorption is expected to increase with pH, particularly for an adsorbate of a cationic nature [103]. Several authors have suggested that at pH values above pH_PZC_, the surface has a net negative charge and tends to accumulate cationic dye molecules due to the electrostatic attraction between the cationic dye molecule and the negatively charged surface or the extrudate [104]. However, MB adsorption is reduced at pH values lower than pH_PZC_ due to the net positive charge on the surface, which causes electrostatic repulsion. As shown in Figure 6, the rate of MB adsorbed improved as pH increased from 3.0 to 9.0. However, the adsorption rate at pH values between 7.0 and 9.0 was relatively lower than that observed at pH values between 3.0 and 7.0. As reported in the literature, the high adsorption capacity observed at alkaline pH values is due to the increase in hydroxyl ions and, therefore, to the increase in electrostatic attraction between the positive and negative charges of the adsorption sites [91]. However, at very alkaline pH levels, it appears that OH ions form a complex with other ions within alkaline pH ranges, which affects the dye–adsorbent interaction [105]. This leads to the precipitation of MB on the adsorbent surface, since the adsorption process is probably a combination of factors, such as electrostatic attraction, adsorption, and precipitation [106].

#### 3.2.2. Adsorption Isotherm

Adsorption isotherm studies show that using the extrudates, the MB removal rate first increases from 0.25 to 20 mg L^−1^, and then decreases when the initial MB concentration (20–30 mg L^−1^) is increased. This can be explained by the fact that at higher concentrations, more MB molecules compete for the active sites available on the surface of the adsorbent material. These active sites, which are limited in amount, quickly become saturated as the concentration of MB increases. Therefore, the initial concentration of dye provides a significant driving force to overcome the mass transfer resistance of the dye between the aqueous solution and the surface of the extrudates [107].

The experimental data of adsorption were fitted to the Langmuir and Freundlich isotherm models. The parameters corresponding to the fit of these results are summarized in Table 3. The correlation coefficients in both isotherm models were close to 1, indicating that the two models fit the experimental data well [108]. However, as shown in Figure 7, the Langmuir isotherm model fit better than the Freundlich isotherm model. It can be concluded that MB adsorption onto these adsorbents can be considered monolayer adsorption rather than multilayer adsorption. This fact supposes that MB adsorption on extrudates occurs as a phenomenon of electrostatic attraction in which the adsorption energy is uniform [109]. During this adsorption process, the cationic dye tends to move through the pores and channels of the extrudates, replacing the exchangeable cations present in the synthesized materials, which are shown in Figure 5.

#### 3.2.3. Adsorption Kinetics

Although the adsorption models help to establish efficiency in the process, it is also important to determine the kinetic mechanism. The adsorption kinetic models express the contact time required for complete adsorption of the chemical species. From them, we can establish the optimal conditions for a process of continuous dye removal and/or scaling at an industrial level. Figure 8 illustrates the MB concentration in an aqueous solution at different contact times. For all adsorbents, it was observed that the MB concentration decreased rapidly at the beginning, and tended to be constant after 60 min. From this trend, we can conclude that equilibrium was reached at the contact time of around 180 min. The rapid initial adsorption stage resulted from the presence of the vacant adsorption sites, as well as from the presence of a high concentration gradient. On the one hand, adsorption by all extrudates can be attributed to the negative surface charge of these materials, which leads to a high electrostatic attraction between the negatively charged sorbents and the positively charged cationic MB [110]. On the other hand, the efficiency of extrudates to adsorb dissolved MB dye molecules is also attributed to the combination of active sites provided by the diatomaceous earth, which acts as a support, and the photocatalysts nanoparticles immobilized on the surface.

The adsorption kinetic parameters are summarized in Table 4. In this study, the highest correlation coefficient (R^2^) was obtained for the pseudo-second-order model, which assumed chemical adsorption o the cationic dye in the extrudates [111]. The adjustment of experimental data to the intraparticle diffusion model shown in Figure 9 allowed for the identification of two linear regions, which suggests that the MB adsorption process could be described by external-film diffusion followed by internal-pore diffusion. Table 4 also summarizes the linear regression analysis for the diffusion kinetic models. The highest values of the regression coefficient (R^2^) were found for the external-film diffusion; furthermore, the values of A were relatively high. Therefore, surface adsorption was the rate-limiting step [112].

### 3.3. Photocatalytic Degradation of MB

It is clear from Figure 10 that the photocatalytic activity of ZTO/La was the highest probably due to it having a high surface area and low bandgap. The results show that the photocatalytic activities of the ZTO/La nanoparticles increased and the bandgap value decreased. This is due to the fact that the energy (*hv*) required is directly proportional to the bandgap and hence reduces the energy needed to excite electrons from the valence band to the conduction band [11].

The use of DE-supported photocatalysts (ZTO-DE and ZTO/La-DE) allowed for an efficient degradation of the MB solution, probably due to the following two main reasons. First, the unique mesoporous structure and higher surface area of DE would significantly improve the adsorption capacity of the material, providing a more active adsorption site towards the target molecules. Second, the incorporation of ZnTiO_3_/TiO_2_ and ZnTiO_3_/TiO_2_/La photocatalysts could facilitate transfer of photogenerated electrons from the bulk to the surface and thus inhibit the recombination of electron pairs and holes under solar irradiation [38]. As is known, under illumination, the electrons of a photocatalyst can be excited and then immediately transferred from the valence band (VB) to the conduction band (CB), generating an electron-hole pair (e^−^/h^+^) and leaving a hole (h^+^) in the VB (reaction R1). The electron-hole pairs can recombine immediately (reaction R2); some of them can also migrate to the surface of the catalyst and react separately with other species adsorbed on the surface, such as H_2_O, OH^−^, O_2_, and other molecules (R), as MB dye. The holes at the semiconductor VB can oxidize adsorbed water or hydroxyl ions to form highly reactive hydroxyl radicals (reactions R3 and R4). On the other hand, the generated electrons at the CB can react with adsorbed oxygen molecules to produce OH radicals via a succession of reactions (reactions R5–R8). These formed hydroxyl radicals have a strong ability to degrade organic dyes such as methylene blue (MB) (reaction R9). Furthermore, direct oxidation of MB could also occur by reaction with holes (reaction R10) [79]. The following reactions represent the probable mechanism of MB photodegradation on the surfaces of ZTO-DE and ZTO/La-DE.

$$\left(semiconductor\right)\overset{hv}{\to }semiconductor+e_{CB}^{-}+h_{VB}^{+}\;(R1)$$

$$e_{CB}^{-}+h_{vb}^{+}\to heat\;(R2)$$

$$H_{2}O_{ads}+h_{VB}^{+}\;⇌\;{\left(H^{+}+OH^{-}\right)}_{ads}+h_{VB}^{+}\to OH_{ads}^{•}\;(R3)$$

$$OH_{ads}^{-}+h_{VB}^{+}\to OH_{ads}^{•}\;(R4)$$

$${\left(O_{2}\right)}_{ads}+e_{BC}^{-}\to O_{2}^{•-}\;(R5)$$

$$O_{2}^{•-}+H^{+}\to HO_{2}^{•}\;(R6)$$

$$2HO_{2}^{•}\to H_{2}O_{2}+O_{2}\;(R7)$$

$$H_{2}O_{2}+e^{-}\to OH^{•}+OH^{-}\;(R8)$$

$$R+OH_{ads}^{•}\to R_{ads}^{ʹ•}+H_{2}O\to degradation\;products\;(R9)$$

$$R_{ads}+h_{VB}^{+}\to R_{ads}^{•+}\to degradation\;products\;(R10)$$

When the ZTO/La is irradiated by solar light, the electrons of La_2_O_3_—which is wrapped on the surface of ZTO—may be excited from ground state to 4*f* orbital. Generally, the photoexcited state of La_2_O_3_ is generated by the absorption of light, corresponding to the transition of the electrons situated in the inner 4*f* orbital to the 5*d* orbitals (4*f*–5*d* transition) or to other 4*f* orbitals (*f*–*f* transition). As a result, the electrons can be freely transported along the surface, leading to better photoelectrochemical and photocatalytic performances of ZTO/La under solar light [39].

### 3.4. Reuse of the Composites

Figure 11 shows the efficiency obtained in the present study for the adsorption and photocatalytic degradation of MB from aqueous solutions. Although DE has low photocatalytic activity, its adsorption capacity is greater and allows for the immobilization of the photocatalyst to facilitate its handling and recovery in the process.

Mechanical stability is an especially important property, which is directly related to the useful life of the supported photocatalyst. When mechanical stability is poor, the photocatalyst will gradually flake away from the support into the reaction solution during the process; consequently, the supported photocatalyst loses its activity prematurely, and causes both secondary contamination and waste of the photocatalyst. Some research results showed that mechanical stability of the material is correlated with the calcination temperature [113]. Consequently, increasing the calcination temperature produces better mechanical stability, although there is an optimal calcination temperature to achieve maximum mechanical stability. In the present paper, a maximum calcination temperature of the extrudates of 500 °C was used to avoid the crystalline phase change of the synthesized photocatalysts. On average, the loss of activity in the materials did not exceed 20% at the end of the fifth cycle. Thus, 500 °C is the optimum calcination temperature to achieve adequate photocatalytic activity and reuse property, under the operating conditions used in this study.

### 3.5. MB Adsorption Capacity and Photocatalytic Activity of the Synthesized Materials Compared to Other Materials Described in the Literature

The results from this paper indicate that diatomaceous earth is a valuable support for photocatalysts, as it contributes with active sites that improve adsorption of dyes for its subsequent photodegradation. Table 5 summarizes the MB adsorption capacity of the synthesized compounds in comparison with other materials reported in the literature.

Similarly, the composites synthesized in this study could be used efficiently to photodegrade dyes in aqueous effluents. Table 6 summarizes the operating and process conditions applied in different research studies that photodegraded MB using various doped TiO_2_. The conditions are described by four factors, including initial MB concentration, type of light used, reaction time, and MB removal efficiency.

Finally, the synthesized DE-supported composite reported in this paper could be an efficient alternative to remove dyes in aqueous effluents and the most probable reason is the combined effects of several factors, such as specific surface area, crystal size and crystallization phases, absorption capacity, photocatalytic activity, and mechanical stability. 

## 4. Material and Methods

### 4.1. Materials

All of the reagents used in this study were of analytical grade and used without additional purification: C_3_H_8_O (Sigma Aldrich, St. Louis, MO, USA, ≥99.5%), Ti(OC_3_H_7_)_4_ (Sigma Aldrich, St. Louis, MO, USA, 98.0%), CH_3_COOH (Sigma Aldrich, St. Louis, MO, USA, 99.8%), H_2_O_2_ (Sigma Aldrich, St. Louis, MO, USA, 35.0%), Zn(CH_3_COO)_2_ · 2H_2_O (ACS, St. Louis, MO, USA, ≥98.0%), C_16_H_18_ClN_3_S · *n*H_2_O (Sigma Aldrich, St. Louis, MO, USA, ≥95.0%), La(NO_3_)_3_ · 6H_2_O (Sigma Aldrich, St. Louis, MO, USA, 99.9%), HCl (Fisher Scientific, Waltham, MA, USA, 37%), cetyl-trimethyl ammonium chloride (C_19_H_42_NCl) (Sigma Aldrich, St. Louis, MO, USA, 25%), AgNO_3_ (Sigma Aldrich, St. Louis, MO, USA, >99.8%), and HNO_3_ (Sigma Aldrich, St. Louis, MO, USA, 69%).

### 4.2. Diatomaceous Earth Purification

The raw diatomaceous earth (DE) was collected from southern Ecuador. The DE sample was ground and sieved to 200-mesh (0.074 mm) size. Calcium and magnesium carbonates were removed using hydrochloric acid (0.1 N) at a ratio of 10 mL g^−1^. The organic matter present in the DE sample was oxidized by adding H_2_O_2_ (33%) at a ratio of 10 mL g^−1^ under agitation for 2 h at room temperature. After centrifugation, the purified DE was washed with distilled water for the removal of Cl^−^ ions; this was checked with a test with AgNO_3_. The DE adsorption sites were activated with nitric acid (0.8 N) in a proportion of 10 mL g^−1^. Activation is a process through which a partially dissolved material is obtained, which has greater surface acidity, porosity, specific surface area, and adsorption capacity [22,23,139,140]. The activated DE samples were centrifuged, washed with distilled water, and dried at 60 °C for 24 h.

### 4.3. Synthesis of the DE-Supported Nanocomposites

The ZnTiO_3_/TiO_2_ (ZTO) and ZnTiO_3_/TiO_2_/La (ZTO/La) nanocomposites were synthesized following a modified sol-gel method described in previous studies [83,90]. To obtain the ZTO nanocomposite, a quantity of titanium (IV) isopropoxide (TiPO) in isopropyl alcohol (iPrOH) (70 *v*/*v*%) was dispersed at room temperature. An aqueous solution formed by Zn(acet), water, and iPrOH was slowly added, using ZnO/TiO_2_ in a 1:3 molar ratio. The amount of water had a 50 *v*/*v*% iPrOH/water ratio and was determined by stoichiometry, being the amount necessary to hydrolyze the TiPO molecules. The synthesis was performed at room temperature. The reaction system was additionally stirred for 30 min. The mixture was kept under stirring at room temperature for another 30 min after formation of a precipitate. The precipitate was dried at 60 °C for 24 h and then calcined at 500 °C for 4 h. Finally, the solids were cooled at room temperature. To obtain the ZTO/La nanocomposite, the procedure described above was repeated, adding La(NO_3_)_3_·6H_2_O to the aqueous Zn solution to obtain a final lanthanum concentration of 1% per gram of ZTO. The previous synthesis process was repeated for each photocatalyst using, at the beginning of the process, a solution (10 *w*/*w*%) of diatomaceous earth in isopropyl alcohol (iPrOH).

### 4.4. Structuring of the DE-Supported Nanocomposites

For the evaluation of the solid materials, cylindrical extrudates with approximate dimensions of 0.2 cm in diameter and 1.0 cm in length were prepared. The preparation of these solids was carried out by mixing each DE-supported nanocomposite with an amount of water (approximately 35%) to form a mixture with good plasticity. This mixture was extruded with a 2.5 mm-diameter syringe. The extrudates were dried at 90 °C for 2 h and finally calcined at 500 °C for 8 h.

### 4.5. Characterization

The synthetized materials were characterized using a JEOL JSM 6400 scanning electron microscope (SEM) (JEOL, Peabody, MA, USA). The X-ray fluorescence (XRF) measurements were recorded in a Bruker S1 Turbo SDR portable spectrometer (Bruker Handheld LLC, Kennewick, WA, USA), using the mining light elements measurement method. The X-ray diffraction (XRD) measurements were recorded in a Bruker-AXS D8-Discover diffractometer (Bruker AXS, Karlsruhe, Germany) equipped with a vertical *θ-θ* goniometer, a parallel incident beam (Göbel mirror), and a HI-STAR general area diffraction detection system (GADDS) (Bruker AXS, Karlsruhe, Germany). The X-ray diffractometer was operated at 40 kV and 40 mA to produce Cu Kα radiation (1.5406 Å). The data were recorded from 5 to 70° in the 2*θ* range. Identification of the crystal phases was obtained by comparison of the XRD profile with the ICDD (International Centre for Diffraction Data, release 2018) database. Determination of the specific surface area of the solids (m^2^/g) was carried out in the ChemiSorb 2720 equipment (Micromeritics, Norcross, GA, USA) by nitrogen adsorption at the temperature of liquid nitrogen (−196 °C) with a 30% gas mixture of N_2_ diluted in He. The Chemisoft TPx system (version 1.03; data analysis software; Micromeritics, Norcross, GA, USA, 2011) allowed the specific surface area to be calculated using the Brunauer–Emmet–Teller (BET) equation and the single-point method. The UV-Vis diffuse reflectance spectrum (DRS) of the photocatalysts was obtained by a UV-Vis Thermo spectrophotometer model: Nicolet Evolution 201/220 (ThermoFisher, Waltham, MA, USA), equipped with an integration sphere unit using BaSO_4_ as reference. Finally, the adsorbents were also characterized by the point of zero charges (pH_PZC_) at different pH values using a Jenway 7350 spectrophotometer (Cole-Parmer, Staffordshire, UK).

### 4.6. Adsorption Studies

The adsorption experiments were conducted using a batch method at room temperature. Typically, 25 mg of extruded adsorbents were magnetically stirred in a methylene blue aqueous solution (100 mL of water containing 20 mg mL^−1^ methylene blue). The remaining concentrations of methylene blue were determined at 623 nm using a Jenway 7350 spectrophotometer (Cole-Parmer, Staffordshire, UK). The adsorption rate of MB was calculated by the absorbance according to the Beer–Lambert law. Samples were drawn at 5 min intervals with a syringe and filtered through a 0.45 µm membrane filter to remove any solid particles interfering with the measurement. All tests were carried out in triplicate. The procedure was repeated using a methylene blue reference solution without extruded adsorbents to eliminate any photolysis effects causing discoloration of the solution due to natural light. The adsorbed quantity *q_e_* of methylene blue was calculated by means of Equation (3):

(3) $$q_{e}=\left(C_{0}-C_{e}\right)\times \frac{v}{w}$$

where *C*_0_ (mg L^−1^) and *C_e_* (mg L^−1^) represent the initial and equilibrium concentration, respectively; *v* (L) is the volume of the solution; and *w* (g) is the mass of the adsorbent [83].

#### 4.6.1. Effect of pH

The effect of pH on MB adsorption onto the adsorbents was investigated under pH values varying from 3 to 10. The initial MB concentration used was 25 mg mL^−1^ for all extrudates. Contact time was fixed at 180 min and corresponded to the time necessary to reach adsorption equilibrium for all adsorbents. To evaluate the impact of pH on the solid surface, a point of zero charges pH_PZC_ measurement was also performed for all extrudates. The pH_PZC_ determinations were performed in aqueous suspensions of the extrudates at two concentrations (0.01 and 0.05 M) of the NaCl inert electrolyte. Potentiometric titrations were made over the entire pH range of 3 to 10.

#### 4.6.2. Isotherm Models

The effect of the initial MB concentration was investigated from 0.25 to 30 mg L^−1^. The experiments were performed without adjusting the pH of the solution. At the end of the experiments, the equilibrium pH was measured and found to be constant, around 7 for each adsorbent. The equilibrium MB adsorption was evaluated according to the Langmuir and Freundlich isotherm models, since these models can help to explain the adsorption mechanism and the heterogeneity of the adsorbent surface [83,117,141,142].

The expression of the Langmuir isotherm model can be represented by Equation (4):

(4) $$\frac{C_{e}}{q}=\frac{1}{K_{L}q_{max}}+\frac{C_{e}}{q_{max}}$$

where *q_max_* is the maximum monolayer adsorption, *K_L_* is the equilibrium Langmuir constant related to the adsorption energy, and *C_e_* is the concentration of solute at equilibrium. Additionally, the *R_L_* separation factor values, which provide an insight into the adsorption nature, can be expressed by means of Equation (5):

(5) $$R_{L}=\frac{1}{\left(1+K_{L}C_{e}\right)}$$

The expression of the Freundlich isotherm model can be represented by Equation (6):

(6) q=KFCe1n

where *K_F_* is the Freundlich constant, which indicates the adsorption affinity of the adsorbents, and 1/*n* is another constant that represents the adsorption intensity.

#### 4.6.3. Kinetic Models

The solute absorption rate of the solute–solution interface was described in this study using reaction-based models, called pseudo-first-order and pseudo-second-order, as well as diffusion-based models, called intraparticle diffusion, external-film diffusion, and internal-pore diffusion [107]. The pseudo-first-order and pseudo-second-order models assume that the difference between the average solid-phase concentration (*q_t_*) and the equilibrium concentration (*q_e_*) is the driving force for adsorption and that the overall adsorption rate is proportional to this driving force. Both equations have been widely applied to explain the experimental results obtained for aqueous pollutants such as dyes and metal ions [83,117,141,142].

The pseudo-first-order kinetic model is expressed by means of Equation (7):

(7) $$ln\left(q_{e}-q_{t}\right)=ln\left(q_{e}\right)-k_{1}t$$

where *k*_1_ is the rate constant (min^−1^) and *q_e_* and *q_t_* represent the MB adsorbed per unit weight (mg g^−1^) at equilibrium and at any time *t*, respectively [59].

The pseudo-second-order kinetic is expressed by means of Equation (8):

(8) $$\frac{t}{q_{t}}=\frac{1}{k_{2}q_{e}^{2}}+\frac{1}{q_{e}}t$$

where *k*_2_ is the pseudo-second-order rate constant (g mg^−1^ min^−1^) [143].

In order to gain good insight into the adsorption mechanism, determination of the rate-limiting step is necessary in the adsorption process. The intraparticle diffusion model, based on the theory proposed by Weber and Morris, assumes that intraparticle diffusion is the rate-control step, which is generally the case for well-mixed solutions [92]. The mathematical expression of the intraparticle diffusion model is described by Equation (9):

(9) $$q_{t}=k_{3}t^{\frac{1}{2}}+A$$

where *k*_3_ (mg g^−1^ min^−1/2^) is the intraparticle diffusion rate constant and A (mg g^−1^) is a constant that indicates the thickness of the boundary layer, i.e., the higher the value of A, the greater the boundary-layer effect. In some cases, the plot *q_t_* versus square root time can show multi-linearity, which indicates that several steps occur in the process. 

The internal-pore diffusion model was also used to describe the kinetic adsorption data. If particle diffusion controls (*D_p_*), the adsorption rate is described using Equation (10):

(10) $$-ln\left(1-{\left(\frac{q_{t}}{q_{e}}\right)}^{2}\right)=\frac{2\pi^{2}D_{p}}{r^{2}}\;t$$

When the adsorption rate is controlled by external-film diffusion, it is expressed by means of Equation (11):

(11) $$-ln\left(1-\left(\frac{q_{t}}{q_{e}}\right)\right)=\frac{D_{f}C_{s}}{h\;r\;C_{z}}\;t$$

where *q_t_* and *q_e_* are the solute loadings on the adsorbent phase at time *t* and at equilibrium (mg g^−1^), respectively; *t* is the contact time (min); *C*_s_ (mg L^−1^) and *C_z_* (mg kg^−1^) are the ion concentrations in the solution and in the adsorbent, respectively; *r* is the average radius of the adsorbent particles (1 × 10^−7^ m); and *h* is the film thickness around the adsorbent particles, accepted as 10^−6^ m for poorly stirred solutions [58]. *D_p_* is the diffusion coefficient in the adsorbent phase (m^2^ min^−1^) and *D_f_* (m^2^ min^−1^) is the diffusion in the film phase surrounding the adsorbent particles.

### 4.7. Photocatalytic Degradation

Heterogeneous photocatalysis experiments were carried out, without adjusting pH = 7.0, for 150 min. Typically, 25 mg of composites were magnetically stirred in a methylene blue (MB) aqueous solution (100 mL of water containing 20 mg L^−1^ methylene blue). The solution was maintained in dark conditions for 30 min to attain the adsorption–desorption equilibrium. The photocatalytic activity of the composites was evaluated by the photocatalytic degradation of methylene blue under solar light radiation. Solar light was simulated by a solar box equipped with an air-cooled 1500 W Xenon lamp (Atlas Material Testing Technology, Mount Prospect, IL, USA), which allows 300–800 nm wavelengths to pass through (ATLAS, SUNTEST CPS+). Irradiance was set to 250 W/m^2^. 

The remaining methylene blue concentrations were determined at 623 nm using a Jenway 7350 spectrophotometer (Cole-Parmer, Staffordshire, UK). The MB removal rate was calculated by absorbance according to the Beer–Lambert law. Samples were drawn at 5 min intervals with a syringe and filtered through a 0.45 µm membrane filter to remove any solid particles interfering with the measurement. All tests were carried out in triplicate using a blank methylene blue solution irradiated with solar light to eliminate any photolysis effect due to the light. 

### 4.8. Reuse of the Supported Photocatalysts

A recycling experiment on photocatalytic degradation of MB by ZTO-DE and ZTO/La-DE was designed to determine the recycling property of these composites. After completing a treatment cycle, the catalyst extrudates were left in quiescent conditions for 60 min to achieve their precipitate. Then, the clear solution was removed from the reaction system and 100 mL of fresh MB solution (5 mg/L) were injected into the reaction system, initiating the next cycle. The recycling experiment was carried out for five cycles. Each cycle lasted 150 min under solar irradiation.

## 5. Conclusions

In summary, according to the results obtained, it can be concluded that the sol-gel method is suitable for preparing La-doped ZTO of nanometric size and with high photocatalytic activity. Diatomaceous earth was effectively used to immobilize the nanocatalyst and incorporate various active sites on the surface of the compound. The supported catalysts were adapted into extrudates and then successfully used for the adsorption and photocatalytic removal of MB in aqueous systems. In general, the experimental adsorption isotherms were fitted to the Langmuir model, which describes monolayer adsorption on a surface containing an indefinite number of identical sites. This model was correlated with the one found in the pseudo-second-order kinetic model, which indicates a chemisorption process in the adsorbent. On the other hand, the La ion exerted a significant effect on the gap band and particle size of the ZTO hybrid catalyst. These physical chemistry changes improved efficiency in the absorption and photodegradation under solar irradiation of MB [144,145].

ZTO/La-DE was found to be highly efficient (96.05%) when compared to ZTO-DE (89.99%) and DE (73.21%) in adsorbing and photodegrading MB dye. In addition, it was observed that composite materials can be recycled up to five times with a total 20% reduction in the MB removal capacity. Finally, the MB removal capacity of the materials synthesized in this study open a door to the potential generation of efficient and ecological technologies that can be used on an industrial scale from available natural resources.

## Figures and Tables

**Table 1:** Composition (*wt*%) of diatomaceous earth.

Al_2_O_3_	SiO_2_	S	P_2_O_5_	K_2_O	CaO	TiO_2_	MgO	Fe_2_O_3_	Co_3_O_4_	SnO_2_	CeO_2_	WO_3_
12.10 (±0.72)	61.00 (±0.80)	0.71 (±0.03)	0.26 (±0.09)	1.19 (±0.02)	0.53 (±0.01)	0.29 (±0.01)	0.06 (±0.00)	1.63 (±0.01)	0.42 (±0.01)	0.16 (±0.04)	0.04 (±0.01)	0.01 (±0.00)

**Table 2:** SSA (m^2^/g) of ZTO/La, ZTO, DE, and composites.

Adsorbent	Form	SSA (m^2^/g)
ZTO/La	Powder	126.45
ZTO	Powder	105.84
DE	Powder	89.84
DE	Extrudate	48.89
ZTO/La-DE	Powder	93.24
ZTO/La-DE	Extrudate	67.38
ZTO-DE	Powder	72.21
ZTO-DE	Extrudate	40.36

**Table 3:** Isotherm parameters for MB adsorption on composites.

Isotherm Parameters		ZTO-DE	ZTO/La-DE	DE
Langmuir	*q_max_* (mg g^−1^)	37.32 (±1.21)	40.44 (±1.06)	77.05 (±2.33)
	*K_L_* (L mg^−1^)	0.63 (±0.10)	0.99 (±0.14)	0.56 (±0.06)
	*R_L_*	0.03	0.02	0.06
	χ^2^	2.27	2.31	2.30
	R^2^	0.99	0.99	0.99
Freundlich	*K_F_* (L mg^−1^)	13.38 (±1.21)	17.24 (±1.51)	20.82 (±2.22)
	*n*	2.85 (±0.90)	3.26 (±0.37)	2.23 (±0.24)
	1/*n*	0.35	0.31	0.45
	χ^2^	6.62	10.52	10.42
	R^2^	0.97	0.96	0.96

**Table 4:** Kinetic parameters for MB removal in composites.

Kinetic Parameters		ZTO-DE	ZTO/LA-DE	DE
Pseudo-first order	*q_max_* (mg g^−1^)	76.21 (±1.37)	75.62 (±1.53)	75.60 (±1.50)
	*k*_1_ (min^−1^)	0.05 (±3.91 × 10^−3^)	0.05 (±4.61 × 10^−3^)	0.06 (±5.49 × 10^−3^)
	χ^2^	14.69	16.83	15.87
	R^2^	0.98	0.98	0.98
Pseudo-second order	*q_max_* (mg g^−1^)	86.35 (±4.40)	85.96 (±1.53)	85.57 (±0.99)
	*k*_2_ (g mg^−1^ min^−1^)	7.11 × 10^−4^ (±6.14 × 10^−5^)	7.63 × 10^−4^ (±7.06 × 10^−5^)	9.41 × 10^−4^ (±5.68 × 10^−5^)
	χ^2^	6.29	6.79	2.89
	R^2^	0.99	0.99	1.00
Intraparticle diffusion	*k*_3_ (mg g^−1^ min^−1/2^)	5.37 (±0.51)	5.66 (±0.52)	6.05 (±0.56)
	*A*	15.31 (±4.37)	14.68 (±4.21)	15.28 (±4.23)
	R^2^	0.87	0.89	0.89
External-film diffusion	*Df* (m^2^ min^−1^)	1.32 × 10^−11^	1.27 × 10^−11^	1.37 × 10^−11^
	R^2^	0.97	0.98	0.93
Internal-pore diffusion	*Dp* (m^2^ min^−1^)	1.20 × 10^−17^	1.24 × 10^−17^	2.00 × 10^−17^
	R^2^	0.99	0.99	0.90

**Table 5:** MB adsorption capacity of synthesized materials and of other materials reported in the literature.

Material	*q_e_* (mg/g)	References
Activated lignin–chitosan composite extrudates	36.25	[114]
TiO_2_/montmorillonite–albumin nanocomposite	18.18	[115]
Carboxymethyl cellulose/ZSM-5/ZIF-8	10.49	[116]
ZSM-5 zeolite	105.82	[117]
NaX zeolite	127.13	[118]
Chitosan/clay microspheres	152.20	[119]
Magnetic chitosan/clay beads	82.00	[120]
Activated carbon–clay composite	178.64	[121]
Hydroxysodalite	10.82	[122]
Kaolin	21.41	[123]
Nonporous silica	91.10	[124]
a-TiO_2_/ZnTiO_3_	16.00	[86]
a-TiO_2_	15.00	[86]
Natural clay	15.40	[125]
Raw coal fly ash	5.06	[126]
Activated carbon	6.43	[127]
DE	77.05	This study
ZnTiO_3_/TiO_2_/DE	37.32	This study
ZnTiO_3_/TiO_2_/La-DE	40.11	This study

**Table 6:** Different operating conditions and efficiency for the photocatalytic oxidation of MB by different doping agents.

Type of Dopant	MB (mg/L)	Type of Light	Reaction Time (min)	Efficiency (%)	Reference
TiO_2_/La	0.1	UV irradiation	120	85	[128]
TiO_2_/Fe	0.1	UV irradiation	120	75	[128]
TiO_2_/La	0.1	Visible irradiation	120	20	[128]
TiO_2_/Fe	0.1	Visible irradiation	120	26	[128]
TiO_2_/Ce	32	Visible irradiation	180	90	[129]
TiO_2_/Au	12	Visible irradiation	48	92	[130]
TiO_2_/Sb	100	Visible irradiation	60	100	[131]
TiO_2_/N	10	Solar light	120	97	[132]
TiO_2_/I	8	Solar light	120	45	[133]
TiO_2_/F	10	Solar light	120	55	[133]
TiO_2_/C	28.5	Visible irradiation	420	70	[134]
TiO_2_/Fe/La	0.1	Visible irradiation	120	91	[128]
TiO_2_/C/N	10	Visible irradiation	180	85	[135]
TiO_2_/N/F	5.74	Visible irradiation	140	16	[136]
TiO_2_/Mn/Fe	10	Visible irradiation	150	85	[137]
ZnTiO_3_/PANI/Ag	10	Visible irradiation	25	96	[138]
ZnTiO_3_/Ag	10	UV irradiation	150	93	[14]
ZnTiO_3_/TiO_2_/La	20	Solar light	150	100	This study
ZnTiO_3_/TiO_2_ (not doped)	20	Solar light	150	87	This study
ZnTiO_3_/TiO_2_/La-DE	20	Solar light	150	93	This study
ZnTiO_3_/TiO_2_-DE (not doped)	20	Solar light	150	85	This study

## Data Availability

Data are available from the authors upon reasonable request.

## Sample Availability

Sample are available from the authors upon reasonable request.
